# Combination of Pembrolizumab with Electrochemotherapy in Cutaneous Metastases from Melanoma: A Comparative Retrospective Study from the InspECT and Slovenian Cancer Registry

**DOI:** 10.3390/cancers13174289

**Published:** 2021-08-25

**Authors:** Luca G. Campana, Barbara Peric, Matteo Mascherini, Romina Spina, Christian Kunte, Erika Kis, Petra Rozsa, Pietro Quaglino, Rowan Pritchard Jones, A. James P. Clover, Pietro Curatolo, Roberto Giorgione, Maja Cemazar, Francesca de Terlizzi, Masa Bosnjak, Gregor Sersa

**Affiliations:** 1 Department of Surgery, The Christie NHS Foundation Trust, Manchester M20 4BX, UK; 2 Department of Surgical Oncology, Institute of Oncology Ljubljana, 1000 Ljubljana, Slovenia; bperic@onko-i.si; 3 Faculty of Medicine, University of Ljubljana, 1000 Ljubljana, Slovenia; 4 Clinica Chirurgica 1, Ospedale Policlinico San Martino, University of Genova, 16100 Genova, Italy; matteo.mascherini@hsanmartino.it; 5 Soft-Tissue, Peritoneum and Melanoma Surgical Oncology Unit, Veneto Institute of Oncology IOV-IRCCS, Padua Veneto Institute of Oncology, 35121 Padova, Italy; romina.spina@iov.veneto.it; 6 Department of Dermatology and Allergology, Ludwig-Maximilian University of Munich, 80539 Munich, Germany; christian.kunte@artemed.de; 7 Department of Dermatologic Surgery and Dermatology, Artemed Fachklinik Munich, 81379 Munich, Germany; 8 Department of Dermatology and Allergology, University of Szeged, 6720 Szeged, Hungary; kis.erika.gabriella@med.u-szeged.hu (E.K.); rozsa.petra@med.u-szeged.hu (P.R.); 9 Dermatologic Clinic, Department of Medical Sciences, University of Turin Medical School, 10124 Torino, Italy; pietro.quaglino@unito.it; 10 Plastic Reconstructive Surgery, University of Liverpool, St. Helens & Knowsley Teaching Hospitals NHS Trust, Liverpool L35 5DR, UK; Rowan.PritchardJones@sthk.nhs.uk; 11 Plastic Reconstructive Surgery, Cork University Hospital and South Infirmary Victoria University Hospital and Plastic Surgery University College Cork, T12 DC4A Cork, Ireland; j.clover@ucc.ie; 12 Policlinico Umberto I, University La Sapienza, 00185 Roma, Italy; Pietro.Curatolo@uniroma1.it; 13 Dermatologic Clinic of University of Eastern Piedmont, 28100 Novara, Italy; giorgions@virgilio.it; 14 Department of Experimental Oncology, Institute of Oncology Ljubljana, 1000 Ljubljana, Slovenia; mcemazar@onko-i.si (M.C.); mbosnjak@onko-i.si (M.B.); 15 Faculty of Health Sciences, University of Primorska, 6310 Izola, Slovenia; 16 IGEA S.p.A., Clinical Biophysics Laboratory Carpi, 41012 Modena, Italy; f.deterlizzi@igeamedical.com; 17 Faculty of Pharmacy, University of Ljubljana, Askerceva 7, 1000 Ljubljana, Slovenia; 18 Faculty of Health Sciences, University of Ljubljana, 1000 Ljubljana, Slovenia

**Keywords:** metastatic melanoma, skin metastases, pembrolizumab, electrochemotherapy

## Abstract

**Simple Summary:**

Electrochemotherapy (ECT) combines a cytotoxic agent with locally applied electric pulses to enhance its antitumor effect. Over the last 15 years, ECT has been safely applied to patients with skin metastases in combination with other oncologic treatments and, more recently, with systemic immunotherapy. In this study, we aimed to investigate the effectiveness of ECT in combination with pembrolizumab. We compared patient outcomes after the following treatments: (a) pembrolizumab, (b) pembrolizumab and ECT, and (c) ECT alone. The combined application of pembrolizumab and ECT was safe and more efficacious in preventing further growth of cutaneous metastases than pembrolizumab alone. The patients treated with pembrolizumab and ECT experienced lower disease progression rates and longer survival than those who received pembrolizumab. ECT may boost the effect of pembrolizumab by acting as an in situ vaccination against cancer cells. Further studies are required to confirm these findings.

**Abstract:**

Electrochemotherapy (ECT) is an effective locoregional therapy for cutaneous melanoma metastases and has been safely combined with immune checkpoint inhibitors in preliminary experiences. Since ECT is known to induce immunogenic cell death, its combination with immune checkpoint inhibitors might be beneficial. In this study, we aimed to investigate the effectiveness of ECT on cutaneous melanoma metastases in combination with pembrolizumab. We undertook a retrospective matched cohort analysis of stage IIIC–IV melanoma patients, included in the International Network for sharing practices of ECT (InspECT) and the Slovenian Cancer Registry. We compared the outcome of patients who received the following treatments: (a) pembrolizumab alone, (b) pembrolizumab plus ECT, and (c) ECT. The groups were matched for age, sex, performance status, and size of skin metastases. The local objective response rate (ORR) was higher in the pembrolizumab-ECT group than in the pembrolizumab group (78% and 39%, *p* < 0.001). The 1 year local progression-free survival (LPFS) rates were 86% and 51% (*p* < 0.001), and the 1 year systemic PFS rates were 64% and 39%, respectively (*p* = 0.034). The 1 year overall survival (OS) rates were 88% and 64%, respectively (*p* = 0.006). Our results suggest that skin-directed therapy with ECT improves superficial tumor control in melanoma patients treated with pembrolizumab. Interestingly, we observed longer PFS and OS in the pembrolizumab-ECT group than in the pembrolizumab group. These findings warrant prospective confirmation.

## 1. Introduction

Recent advances in the treatment of metastatic melanoma have been achieved with the introduction of BRAF and MEK inhibitors, followed by immunotherapy with immune checkpoint inhibitors (ICIs). Humanized monoclonal antibodies such as anti-CLA-4 (ipilimumab) and anti-PD1 (pembrolizumab and nivolumab) release the inhibitory brake on T cells, unleashing the body’s immune response against cancer [1,2]. Together with targeted therapy, ICIs are now the standard of care for patients with metastatic melanoma [3]. Yet, almost 40–60% of them fail to respond [4]. A recent analysis of 583 melanoma patients treated with pembrolizumab as part of the KEYNOTE-001 study identified LDH, site of metastases, and no prior therapy as the predictor of response, whereas LDH, low tumor burden, site of metastases, and ECO-PS were identified as predictors of OS [5].

Approximately 8–10% of patients with stage IIIC and IV disease develop skin metastases in the form of either in-transit or distant metastases [6]. Historically, several therapeutic approaches have been developed to treat these patients. Available options include locoregional chemotherapy (hyperthermic isolated limb perfusion (HILP), isolated limb infusion (ILI)), radiation, and several lesional therapies (Rose Bengal (PV-10), oncolytic virotherapy (T-VEC), imiquimod, and diphencyprone (DCP)) [7,8,9,10,11]. More recently, systemic immunotherapy and, in BRAF V600E/K-mutated melanoma, targeted therapy have been introduced with appreciable results [12].

Electrochemotherapy (ECT) has entered into the armamentarium of skin-targeted therapies only recently; however, thanks to the progressive accumulation of a solid evidence basis on its efficacy and tolerability, it has been included in the current ESMO melanoma guidelines [13,14,15]. In ECT, tumor electroporation provides a highly efficient delivery system for two inexpensive agents, i.e., bleomycin and cisplatin. By destabilizing the cell membrane, electroporation enables drug diffusion into cells thus potentiating the cytotoxic effect locally [14]. The efficacy of ECT in melanoma has been demonstrated by several studies [16,17,18]. A recent analysis of the International Network for Sharing Practice in Electrochemotherapy (InspECT) database, a prospective registry including 987 patients with skin metastases (283 from melanoma), indicated an overall response rate (ORR) of 82% with a CRR of 64% [19]. Interestingly, preliminary experiences suggest that ECT may elicit an immunogenic cell death, thus functioning as a form of in situ vaccination. Therefore, its combination with either ICIs or other forms of immunotherapy is of potential interest [20,21]. Furthermore, in preclinical studies, the in situ vaccination effect of ECT was proven, which can be boosted by adjuvant immunogenic therapy [20,21,22,23]. The first clinical experiences have demonstrated the feasibility and safety of the combination of ICIs with ECT [24].

The aim of this study was to evaluate the local response and tumor control on cutaneous metastases in melanoma patients who received pembrolizumab and ECT. Moreover, we assessed toxicity, systemic disease progression, and survival.

## 2. Materials and Methods

### 2.1. Patient Selection

Data of patients with histologically proven stage IIIC–IV melanoma with measurable cutaneous metastases suitable for ECT application were collected for this study. Patient demographics, disease stage, location, number and size of skin metastases, previous treatments, ECT parameters, and toxicity were recorded. The study cohort comprised three groups of patients who received the following treatments: (a) pembrolizumab plus ECT, (b) pembrolizumab alone, and (c) ECT alone. The data of the pembrolizumab–ECT and ECT groups were retrieved from the InspECT database, while those of the pembrolizumab cohort were collected from the Clinical Registry of Skin Melanoma (CRRS).

### 2.2. InspECT Database

The InspECT group is a European collaborative including 41 clinical centers applying ECT. In this study, we analyzed data from 86 patients treated at 10 institutions between 2011 and 2019. The registry collects baseline patient and tumor characteristics, ECT modalities, treatment toxicity, and patient outcome. Ethics approval was sought by each institution individually. Participation in the database collaboration was by signed agreement [25]. Patient referral for ECT was agreed following multidisciplinary discussion. For patients who received combined treatment with pembrolizumab and ECT, additional information on immunotherapy was collected, including response according to the immune-related response criteria. As an inclusion criterion, in the combined group, ECT had to be performed after the start of immunotherapy or within 3 months of its initiation. Data of patients receiving the combined treatment were matched to patients treated with ECT alone. The matching variables were (a) sex, (b) age, (c) Eastern Cooperative Oncology Group (ECOG) performance status PS, (d) and tumor size of skin metastases.

### 2.3. Clinical Registry of Skin Melanoma (CRRS)

The control group was a cohort of 44 stage IIIC or IV patients treated with pembrolizumab from 2016 until 2020 retrieved from the CRRS (part of The Cancer Registry of Slovenia). These patients were matched with the InspECT registry cohorts for (a) sex, (b) age, (c) ECOG PS, and (d) tumor size.

### 2.4. Treatment

ECT was performed according to the European Standard Operating Procedures of ECT (ESOPE) [26]. Bleomycin was administered either intratumorally (i.t.) using 1000 IU/mL or intravenously (i.v.) using 15,000 IU/m^2^. The route of administration was selected depending on the number of cutaneous metastases and their size. Depending on tumor characteristics, one of the following electrode geometries was used: plate, row needle, or hexagonal needle. Electric fields (eight pulses of 100 μs duration, 5 kHz repetition frequency) were delivered using a square wave electric pulse generator (IGEA, Carpi, Italy).

### 2.5. Response Evaluation

Local response was evaluated at 6 months after ECT, adapting the Response Evaluation Criteria in Solid Tumors (RECIST version 1.0) to assess cutaneous metastases. Complete response (CR) was defined as the disappearance of all target lesions, whereas partial response (PR) was defined as at least a 30% decrease in the sum of the largest diameters. Progressive disease (PD) was defined as a 20% increase in size; stable disease (SD) response had neither sufficient shrinkage to qualify for PR nor sufficient increase to qualify for PD. A maximum of seven cutaneous metastases (including the largest tumor) were registered as target lesions [27,28]. Systemic response was assessed at 6 months after ECT in the pembrolizumab–ECT group and 6 months after immunotherapy initiation in the pembrolizumab group according to the immune-related response criteria (irRC) [29] using PET-TC, TC, MR, and bone scintigraphy. Accordingly, irCR was the disappearance of all tumors and no appearance of new lesions, with confirmation at 4 weeks; irPR was a >50% decrease of tumor burden; irSD was a response not meeting the criteria for irCR or irPR in the absence of irPD; irPD was an >25% increase in tumor burden.

### 2.6. Statistical Analysis

Statistical analysis was conducted with NCSS software (Hintze, J. (2013). NCSS 9. NCSS, LLC., Kaysville, UT, USA. www.ncss.com; access on 26 October 2020). Continuous variables are described by median value and range, whereas categorical variables are described by absolute number and percentage. The analysis of the response for different groups was reported using 2 by k contingency tables and analyzed by the chi-square test. Local tumor control is expressed as local progression-free survival (LPF), which was the time from the first ECT or immunotherapy initiation until local relapse, progression, or last follow-up (whichever came first). Similarly, systemic progression-free survival (PFS) was the time from the first ECT or immunotherapy initiation to systemic relapse, progression, or last follow-up (whichever came first). Overall survival (OS) was calculated from the first ECT or immunotherapy initiation until death or last follow-up. Survival curves were calculated using the Kaplan–Meier method. Cox regression analysis was performed to investigate the survival function corrected for previous systemic therapies and corrected risk ratio was reported with 95% CI. Tests were two-sided. A value of *p* < 0.05 was considered statistically significant.

## 3. Results

### 3.1. Patients

The study cohort (*n* = 130) included three subgroups according to the received treatment: pembrolizumab plus ECT (*n* = 45), pembrolizumab alone (*n* = 44), and ECT alone (*n* = 41). Patient characteristics are reported in Table 1.

There was a significant difference among groups for previous local therapies and systemic treatment (Table 1). The median interval between the initiation of immunotherapy and ECT was 2.4 months (range, from −3 to 41 months). The median time between diagnosis and ECT in the pembrolizumab-ECT group was 2.8 years (0.6–10.4), and, in the ECT group, it was 2.0 years (0.3–22.3) (*p* = 0.180). Only two patients received the first ECT treatment before the start of immunotherapy, at 89 and 5 days.

### 3.2. Toxicity

No serious adverse events were reported. The side-effects are listed in Table 2. In the pembrolizumab group, all cases of pneumonitis with one exception were successfully managed and resolved.

### 3.3. Local Tumor Response

Local response was significantly higher among patients who received ECT—alone or with pembrolizumab—when compared to pembrolizumab alone (Table 3). No differences in response could be observed between patients who underwent intralesional or intravenous administration of bleomycin (*p* = 0.601).

The analysis of patients with local progression and time to local progression favored the pembrolizumab–ECT group over the pembrolizumab group (Table 3). 

### 3.4. Systemic Response

Systemic response in the pembrolizumab–ECT and pembrolizumab groups is reported in Table 4.

The systemic ORR was similar between groups. The number of patients who had systemic progression was similar, but the time to systemic progression, as well as the systemic control of the disease, was significantly longer in the pembrolizumab–ECT group than in the pembrolizumab group (Table 4).

### 3.5. Survival

**Figure 1:**
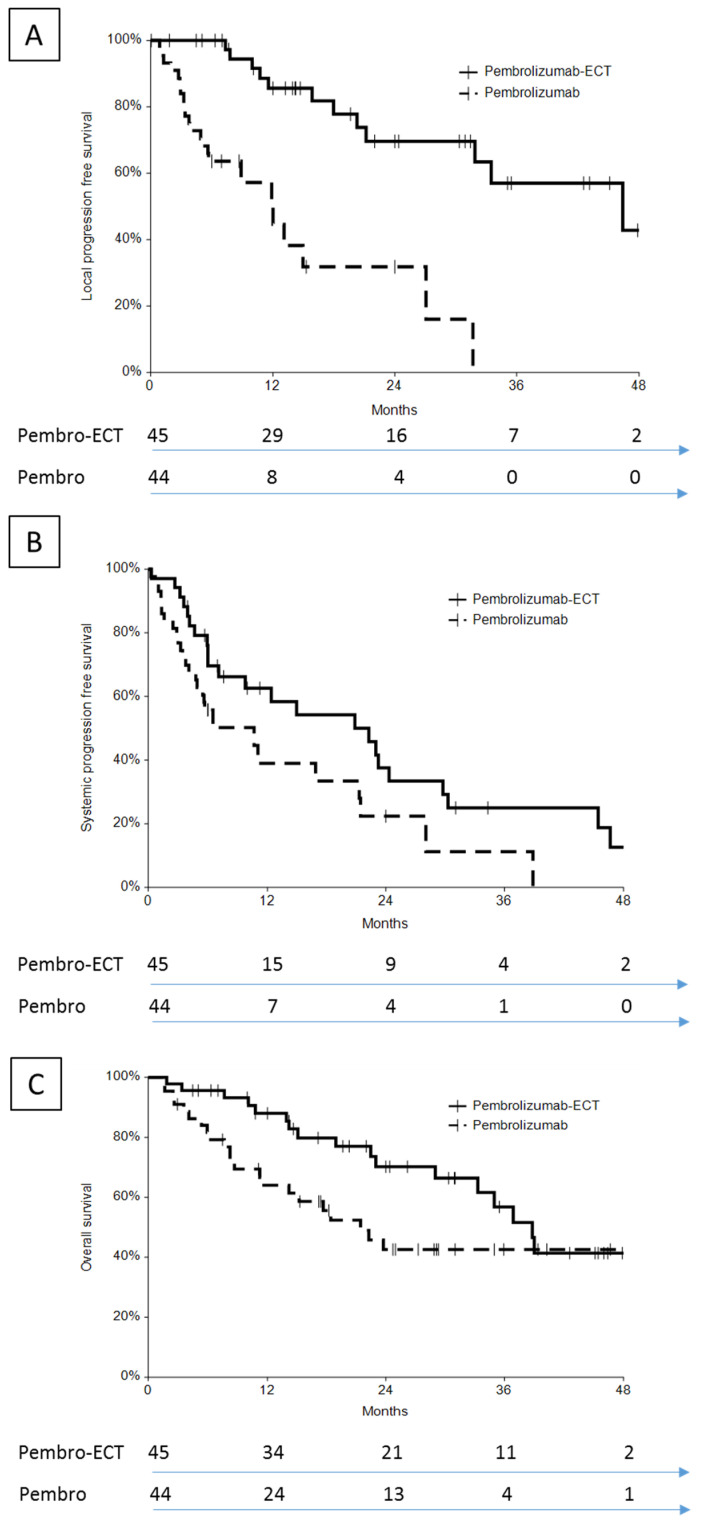
Outcome of patients treated with pembrolizumab–ECT or pembrolizumab alone: (**A**) local PFS; (**B**) systemic PFS; (**C**) OS. The number of patients at risk is indicated on the bottom.

The patients in the pembrolizumab–ECT group had better local PFS, systemic PFS, and OS when compared to the pembrolizumab group (Figure 1). The difference in outcome between groups was confirmed after applying a correction for previous systemic treatment (Table 5). 

#### 3.5.1. Local Control

The 1 year local PFS was 86% (74–97%) in the pembrolizumab–ECT group and 51% (31–70%) in the pembrolizumab group; the 2 year local PFS was 70% (53–87%) and 32% (11–53%), respectively.

Local tumor control was significantly more durable in the pembrolizumab–ECT compared to the ECT group (median local PFS, not reached vs. 14 months, *p* = 0.029; Figure S1).

#### 3.5.2. Systemic Control

The 1 year systemic PFS was 64% (46–80%) in the pembrolizumab–ECT group and 39% (20–58%) in the pembrolizumab group; the 2 year systemic PFS was 38% (19–56%) and 22% (4–40%), respectively.

#### 3.5.3. Overall Survival

The 1 year overall survival (OS) was 88% (78–98%) in the pembrolizumab–ECT group and 64% (49–79%) in the pembrolizumab group; the 2 year OS was 70% (55–85%) and 43% (26–59%), respectively.

## 4. Discussion

In this exploratory analysis, skin-directed treatment with ECT in melanoma patients treated with pembrolizumab proved to be a safe and effective therapy to improve tumor response and local control on cutaneous metastases. Interestingly, our data also show improved disease PFS and OS in the pembrolizumab–ECT group compared to pembrolizumab alone.

### 4.1. Selection of Patients

The application of ECT in conjunction with immunotherapy is not new [30]. To date, however, only four reports are available on its combination with ICIs, including two case reports and two retrospective series [24,31,32,33]. In 2016, Heppt et al. performed a retrospective analysis of 33 highly pretreated stage III–IV melanoma patients who received systemic immunotherapy, with either anti-CTLA-4 (*n* = 28) or anti-PD1 (*n* = 5) agents, combined with ECT. The combined approach proved to be feasible and tolerable and was associated with improved response rate, although severe systemic adverse events were observed in 25% of patients in the ipilimumab plus ECT group [24]. 

Considering these findings and the prevalent use of pembrolizumab in current clinical practice, we decided to assess the outcome of patients treated with pembrolizumab and ECT in the InspECT database. As a control group, we selected a cohort of matched patients from the Slovenian Cancer Registry. It is worth noting that the patients in the pembrolizumab–ECT group were more heavily pretreated when compared to the pembrolizumab group. As the extensive use of previous systemic treatment is expected to be associated with poorer patient prognosis, this bias was in favor of the pembrolizumab group. Nonetheless, we corrected this imbalance using the Cox regression analysis (Table 5). Unfortunately, the relatively small number of patients made it impossible to include further matching variables. 

### 4.2. Safety

One important question is whether the addition of ECT to pembrolizumab may result in exacerbation of toxicity. In this regard, previous reports did not raise specific concerns, especially with anti-PD1 agents [24,31,32,33]. Overall, no serious adverse events were reported in our study, except for one case of pneumonitis in the pembrolizumab group. Moreover, there were no significant differences in terms of local toxicity among the three groups, indicating that pembrolizumab does not add to ECT local side-effects. Nonetheless, it is crucial that clinicians continue to remain vigilant regarding the potential for overlapping toxicity between ICIs and bleomycin [14]. In this regard, it is worth remembering the overall incidence of ICI-related interstitial lung disease ranges between 1% and 4% in melanoma studies [34].

### 4.3. Local Effectiveness

There is a lack of solid data about the effectiveness of immunotherapy on cutaneous metastases, likely due to the scarce representation of patients with stage III in-transit and stage IV–M1a disease in the major randomized trials [35]. In addition, owing to the remarkable results of immunotherapy in terms of survival, local tumor control on cutaneous metastases is underreported. Nonetheless, local tumor control, particularly on superficial disease, remains an important issue to address with the aim of preserving patient quality of life. In the present study, ECT was found to be equally effective in the pembrolizumab–ECT group and in the ECT group (ORR, 77.8% and 80.5%, respectively). However, interestingly, the patients who received the combined treatment had an improved local control on skin metastases (Figure S1). Taken together, these findings suggest considering the adjunct of systemic immunotherapy in patients with skin metastases treated with local therapy with the goal to consolidate tumor control locally. 

Another interesting finding from this study is the contribution of local treatment with ECT in patients treated with ICIs. Of note, local response on cutaneous metastases was significantly higher in the pembrolizumab–ECT than in the pembrolizumab group (ORR 77.8% vs. 56.1%). This indicates that ECT may contribute to improving local tumor control, as also indicated by our positive data on local PFS. Based on the significant improvement of local control, we believe that ECT should be considered as a safe and effective adjunct to the standard treatment of patients with persistent or nonresponsive cutaneous metastases. 

### 4.4. Does ECT Contribute to a Systemic Response?

Next, we aimed to assess whether local treatment with ECT might influence systemic response and patient outcome. A synergistic systemic effect of the combined treatment approach was observed in preliminary experiences [31,32]. In a case report, Brizio et al. reported a systemic response on visceral and distant cutaneous metastases following combined treatment with pembrolizumab and ECT. Mozzilo et al. [32] and Heppt et al. [24] reported systemic ORRs of 60% and 40%, respectively, following a combination of ECT and anti-CTLA4 or anti-PD1 treatment. These studies pose the question of whether ECT might contribute to increase tumor response on distant metastases in patients receiving ICIs. The underlying hypothesis is that ECT acts as an in situ vaccination, whereas immunotherapy (with ICIs or IL-12) elicits a systemic immune response [20,22]. The combination of IL-12 plasmid electroporation and electrochemotherapy and PD-1 blockade is currently under active investigation [23,36,37]. ICIs play a crucial role in the modulation of the antitumor immune response. Tumors are now considered immunologically hot or cold, with the immune score providing strong prognostic capability [38,39,40,41,42]. ECT has been shown to induce an immunogenic cell death and increase immune cells infiltration in the tumor environment, thus potentially turning non-inflamed “cold” tumors into “hot” ones [2,43,44]. Therefore, ECT could render tumors more responsive to ICIs, thus improving both local and systemic response. 

The results of this study indicate that ECT improves the local PFS of patients receiving checkpoint inhibition with pembrolizumab. Additionally, we observed a longer systemic PFS and OS in the combined treatment group. Although our type of study cannot provide any causative correlation, the better outcome of patients in the combined treatment group suggests the involvement of a systemic immune response. The administration of local therapies before checkpoint inhibition has attracted interest due to the possibility of eliciting a strong local immune stimulation on which to capitalize with ICIs. This approach needs to be explored in future clinical and translational research [21].

### 4.5. Why and How to Combine Local Therapies with Immunotherapy

The availability of several skin-directed therapies, coupled with the improved outcomes observed with systemic treatment in patients with stage III in-transit melanoma, poses a therapeutic dilemma regarding the best therapeutic strategy. In this context, the clinical decision making includes three options, i.e., local therapy, systemic therapy, and local therapy variably combined/sequenced with systemic treatment. Which patients are best suited for each strategy is a matter of debate [9,45]. In clinical practice, several variables should be taken into consideration, including patient desires, ECOG-PS, comorbidities, disease stage, response to previous treatment, and number and spread of skin metastases. In our study, we observed that the adjunct of pembrolizumab significantly improved local control on skin metastases (Figure S1). These findings suggest that, although ECT is associated with high response rates, its long-term results can be considerably improved by associating a checkpoint inhibitor. 

Recently, ICIs have been introduced in the treatment of several other histotypes, including lung, urothelial, gastric, and head and neck malignancies [2], where the combination with ECT may prove to be beneficial. In terms of feasibility, thanks to the most recent technical advancements (i.e., *variable-geometry* ECT), ECT can also be delivered to deep-seated tumors [46]. However, before moving forward, more insights into the biological bases of ECT are needed to establish which patients may benefit most from this combined treatment approach [21]. Lastly, the local efficacy of ECT itself may be potentiated by the combined administration of IL-12 gene therapy [23].

### 4.6. Study Limitations

We acknowledge the retrospective nature of this study and the accrual of patients from different databases with the potential limitations inherent to registry-based research. Moreover, the relatively small number of patients in each cohort prevented the inclusion of some clinically important variables among the matching parameters. Lastly, it should be noted that the better outcome of the patients in the pembrolizumab–ECT group may have been influenced by the relatively lower proportion of subjects with visceral metastases compared to pembrolizumab group (42% vs. 56%, Table 1). 

## 5. Conclusions

Local treatment with ECT improves local response and tumor control on cutaneous metastases in melanoma patients receiving anti-PD1 immunotherapy with pembrolizumab, with no toxicity concerns. Interestingly, we also observed prolonged systemic disease control and survival in the combination group when compared to pembrolizumab alone. Conversely, the addition of pembrolizumab in patients treated with ECT alone was found to significantly increase local control on skin metastases. 

These findings suggest a synergistic interaction between these treatment modalities, where ECT might improve patient outcome by acting as an in situ vaccination, boosting the ICI response. These findings prompt additional prospective investigations to confirm the results and clarify the underlying biologic mechanisms. 

## Figures and Tables

**Table 1:** Patient characteristics.

Variable	Pembrolizumab–ECT	Pembrolizumab	ECT	*p*
No.	45	44	41	
Sex				0.723
M	25 (56%)	22 (50%)	24 (60%)	
F	20 (44%)	22 (50%)	17 (40%)	
Age				0.132
median (range)	67 (32–87)	66 (28–82)	70 (44–85)	
Disease stage °				0.285
IIIC	16 (35.6%)	9 (20.5%)	12 (29.3%)	
IV	29 (64.4%) ^†^	35 (79.5%) ^‡^	29 (70.7%)	
Previous local therapies				<0.001
No	15 (33.3%)	27 (61.4%)	5 (12.2%)	
Yes	30 (66.7%)	17 (38.6%)	36 (73.2%)	
Surg	14 (31.1%)	1 (2.3%)	29 (70.7%)	
Surg + RT	11 (24.4%)	3 (6.8%)	4 (9.8%)	
Surg + ILP	4 (8.8%)	1 (2.3%)	0 (0%)	
Surg + RT + ILP	0 (0%)	0 (0%)	1 (2.4%)	
Other	1 (2.2%) *	12 (27.3%) **	2 (4.9%) ***	
Previous Systemic Therapies				0.004
No	15 (33.3%)	30 (68.2%)	19 (46.3%)	
Yes	30 (66.6%)	14 (31.8%)	22 (53.7%)	
CT	3 (6.7%)	1 ((2.3%)	7 (17.1%)	
TT	2 (4.4%)	10 (22.7%)	3 (7.3%)	
IT	14 (31.1%)	3 (6.8%)	8 (19.5%)	
CT + IT	6 (13.3%)	0 (0%)	1 (2.4%)	
CT + TT	0 (0%)	0 (0%)	1 (2.4%)	
IT + TT	3 (6.7%)	0 (0%)	1 (2.4%)	
CT + IT + TT	2 (4.4%)	0 (0%)	1 (2.4%)	
ECOG PS				0.259
0	28 (62.2%)	20 (45.5%)	28 (68.3%)	
1	11 (24.4%)	15 (34.1%)	7 (17.1%)	
2	6 (13.3%)	9 (20.5%)	6 (14.6%)	
Tumor size				0.187
<1 cm	3 (6.7%)	7 (15.9%)	3 (7.3%)	
1–3 cm	22 (48.9%)	27 (61.4%)	22 (53.7%)	
>3 cm	20 (44.4%)	10 (22.7%)	16 (39.0%)	
Anatomical location				0.057
Head/Neck	6 (13.3%)	8 (18.2%)	8 (19.5%)	
Chest	4 (8.9%)	16 (36.4%)	3 (7.3%)	
Abdomen	2 (4.4%)	1 (2.3%)	1 (2.4%)	
Back	4 (8.9%)	4 (9.1%)	6 (14.6%)	
Perigenital	2 (4.4%)	0 (0%)	3 (7.3%)	
Gynecological	1 (2.2%)	0 (0%)	0 (0%)	
Upper limb	2 (4.4%)	2 (4.5%)	1 (2.4%)	
Lower limb	24 (53.3%)	13 (29.5%)	19 (46.3%)	
No. of ECT sessions				0.175
1	28	-	33	
2	13	-	6	
3	4	-	2	

Disease stage was recorded at the time of immunotherapy initiation in the pembrolizumab–ECT and pembrolizumab groups, whereas it was recorded at the time of ECT application in the ECT group. ^†^ stage IV-M1a, *n* = 10; stage IV-M1c-d, *n* = 19. ^‡^ stage IV-M1a, *n* = 10; stage IV-M1c-d, *n* = 25. * ECT (*n* = 1); ** ECT (*n* = 3), RT (*n* = 4), ILP (*n* = 4), ECT + ILP + RT (*n* = 1), *** topical (*n* = 1), laser (*n* = 1). CT = chemotherapy, ECT = electrochemotherapy, ILP = isolated limb perfusion, IT = immunotherapy, RT = radiotherapy, TT = targeted therapy.

**Table 2:** Toxicity.

Toxicity	Pembrolizumab–ECT (*n* = 45)	Pembrolizumab (*n* = 44)	ECT (*n* = 41)	*p*
Odor	5 (11%)	0	3 (7%)	0.716
Suppuration	8 (18%)	0	4 (10%)	0.358
Ulceration	9 (20%)	7 (16%)	12 (29%)	0.311
Hyperpigmentation	9 (20%)	0	9 (22%)	1.000
Nausea	7 (16%)	0	0	0.012
Pneumonitis	2 (4%)	4 (9%)	0	0.434
Pruritus	2 (4%)	1 (2%)	0	1.000
Hypothyroidism	1 (2%)	2 (5%)	0	0.616
Pancreatitis	0	1 (2%)	0	0.494
Colitis	1 (2%)	1 (2%)	0	1.000
Vitiligo	0	1 (2%)	0	0.494

**Table 3:** Local response on skin metastases and disease control.

Response	Pembrolizumab–ECT	Pembrolizumab	ECT	*p*
CR	22 (48.9%)	14 (31.8%)	18 (43.9%)	
PR	13 (28.9%	3 (6.8%)	15 (36.6%)	
SD	7 (15.6%)	13 (29.5%)	7 (17.1%)	
PD	3 (6.7%)	12 (27.3%)	1 (2.4%)	
NE	0 (0%)	2 (4.5%)	0 (0%)	
OR	35 (77.8%)	17 (38.6%)	33 (80.5%)	<0.001
No of pts with local progression	12 (26.7%)	23 (56.1%)	11 (26.2%)	0.016
Time to local progression (months)	20 ± 12	7 ± 8	5 ± 4	<0.001
Time to disease progression (months)	22 ± 15	8 ± 7	-	<0.001

CR = complete response, PR = partial response, SD = stable disease, PD = progressive disease, NE = not evaluable, OR = objective response CR + PR.

**Table 4:** Systemic response (immune-related criteria).

Response	Pembrolizumab–ECT	Pembrolizumab	*p*
CR	5 (11.1%)	9 (20.5%)	
PR	6 (13.3%)	2 (4.5%)	
SD	10 (22.2%)	3 (6.8%)	
PD	17 (37.8%)	30 (68.2%)	
NA	7 (15.6%)	0 (0%)	
OR	11 (24.4%)	11 (25%)	1.000
No of pts in systemic progression	24 (53.3%)	27 (61.4%)	0.522
Time to systemic progression (months)	17 ± 15	8 ± 9	<0.001
Systemic control of disease (months)	17 ± 16	8 ± 8	0.001

**Table 5:** Kaplan–Meier hazard risk evaluation and Cox regression analysis with risk ratio evaluation corrected for previous systemic treatment.

Pembrolizumab–ECT vs. Pembrolizumab (*n* = 45) (*n* = 44)	Kaplan–Meier			Cox Regression (with Correction for Previous Systemic Therapies)		
	HR	95% CI	*p*	RR	95% CI	*p*
Local PFS	4.38	2.13–8.99	<0.001	5.76	2.41–13.77	<0.001
						
Systemic PFS	1.70	1.07–3.60	0.042	1.06	1.07–3.60	0.030
						
OS	1.80	0.95–3.40	0.063	2.02	1.01–4.03	0.046

## Data Availability

The data presented in this study are available on request from the corresponding author. The data are not publicly available due to the privacy of the patients.

## Supplementary Materials

The following are available online at https://www.mdpi.com/article/10.3390/cancers13174289/s1, Figure S1: Kaplan-Mayer curve for local progression-free survival (LPFS) in patients treated with ECT alone and ECT plus pembrolizumab.
