# Supplementary material for: Combination of Pembrolizumab with Electrochemotherapy in Cutaneous Metastases from Melanoma: A Comparative Retrospective Study from the InspECT and Slovenian Cancer Registry
Source: Cancers (Basel). 2021 Aug 25;13(17):4289. doi: 10.3390/cancers13174289 (PMC8428335; doi:10.3390/cancers13174289)
Supplement: Supplementary file 1 [file cancers-13-04289-s001.zip › cancers-1309227-supplementary.pdf]

cancers

MDPI

Article

Combination of Pembrolizumab with Electrochemotherapy in Cutaneous Metastases from Melanoma: A Comparative Retrospective Study from the InspECT and Slovenian Cancer Registry

Luca G. Campana, Barbara Peric, Matteo Mascherini, Romina Spina, Christian Kunte, Erika Kis, Petra Rozsa, Pietro Quaglino, Rowan Pritchard Jones, A. James P. Clover, Pietro Curatolo, Roberto Giorgione, Maja Cemazar, Francesca de Terlizzi, Masa Bosnjak and Gregor Sersa

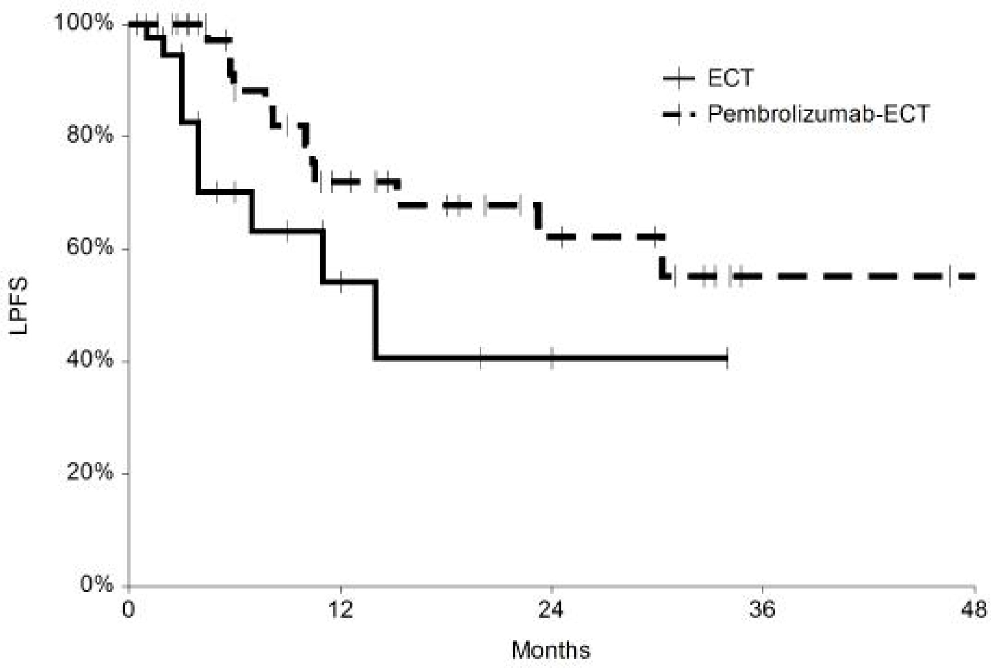

Figure 1. Kaplan-Mayer curve for local progression-free survival (LPFS) in patients treated with ECT alone and ECT plus pembrolizumab. LPFS was the interval between ECT application and in-field recurrence or progression. Median LPFS was 14 months vs. not reached, respectively, p = 0.029. .
